# Letter to the editor: Regarding the editorial by Penttinen and Friede

**DOI:** 10.2807/1560-7917.ES.2016.21.40.30366

**Published:** 2016-10-06

**Authors:** Alicia M Fry, Brendan Flannery, Sonja J Olsen, Lisa Grohskopf, Joseph Bresee

**Affiliations:** 1 Influenza Division, National Centers for Immunization and Respiratory Diseases, Centers for Disease Control and Prevention, Atlanta, United States

**Keywords:** influenza, influenza virus, vaccines and immunization

**To the editor:** In the editorial by Penttinen and Friede, the authors summarised data from 2015 to 2016 on live attenuated influenza vaccine (LAIV) effectiveness in a Table and used the data in the Table to make several conclusions [1]. Unfortunately, the Table has several errors and, therefore, misrepresents the available data and studies. On 26 June 2016, the United States (US) Advisory Committee for Immunization Practices (ACIP) recommended that LAIV not be used during the 2016/17 season in the US [2]. Among all studies using a test-negative case–control design (TNCCD), the study from the US Centers for Disease Control and Prevention (CDC) (US Influenza Vaccine Effectiveness (VE) network) and the US Department of Defence (DoD) study (US Air Force School of Aerospace Medicine (USAFSAM) Sentinel Provider network) had the largest number of influenza A(H1N1)pdm09-infected children aged 2–17 years and larger or comparable numbers of children who received LAIV; these numbers were not correctly shown in the Table in the editorial. The US CDC study included 133 children aged 2–17 years who received LAIV (23 LAIV-vaccinated children had influenza A(H1N1)pdm09 infection) and 1,078 children who were unvaccinated. The US DoD study included 93 children vaccinated with LAIV (23 LAIV-vaccinated children had influenza A(H1N1)pdm09 infection) and 338 unvaccinated children (personal communication September 2016, Susan Federinko, USAFSAM). The youngest age for which LAIV is licensed for use in the US is two years; the US CDC VE estimates refer to children aged 2–17 years. The sample sizes for the other studies in the Table should also be consistently reported so that the same numerical comparisons are available for each study.

The authors incorrectly reported the lower confidence interval of the influenza A(H1N1)pdm09 VE estimate from the study in the United Kingdom (UK) as 8.5 instead of −8.5 [3]. They also incorrectly suggested in the text that this VE result was statistically significant, when it was not significant. Also, the VE estimate from Finland was for type A influenza, not for influenza A(H1N1pdm09). Thus, all studies that included an RT-PCR-confirmed H1N1pdm09 virus outcome failed to find statistically significant protection against influenza A(H1N1)pdm09 infection by LAIV. Conversely, all studies found significant protection against influenza A(H1N1)pdm09 infection for inactivated influenza vaccines (IIV) and reported higher point estimates for IIV [2,3]. In fact, US children who received LAIV were three times more likely to be influenza A(H1N1)pdm09 vaccine failures than children who received IIV during 2015/16 [2]. Data from the previous five influenza seasons in the US, and all other data from the US (ICICLE, DoD) and other countries that were available at that time, were used to inform the 26 June 2016 ACIP decision and the subsequent decision by the American Academy of Paediatrics [2,4,5]; both of these interim decisions are aimed at maximising the likelihood that influenza vaccination will protect US children in the upcoming season.

As Penttinen and Friede state, studies before the 2009 influenza pandemic suggested that LAIV was efficacious and offered some advantages over IIV in young children [1]. Also, some recent studies have suggested a role for LAIV in strategies to immunise against poorly immunogenic novel avian influenza viruses. Antibody titres after vaccination with either IIV or LAIV pre-pandemic avian influenza vaccines were suboptimal, even with higher antigen doses [6]. However, monovalent LAIV effectively primed for a protective antibody response to a single booster dose of IIV containing a matched or related haemagglutinin [6]. Thus, LAIVs have a role in strategies to prevent both seasonal and pandemic influenza infections. It is critical to understand why LAIV did not work as expected against the 2009 pandemic virus in the multivalent formations. In addition, information on the effects of prior vaccination on LAIV vaccine effectiveness will be critical since US children have high influenza vaccine coverage and many are vaccinated with IIV before the age of two years. This information will improve future influenza LAIVs and enhance our ability to utilise them optimally.
